# A modified approach to recover and enumerate *Ascaris* ova in wastewater and sludge

**DOI:** 10.1371/journal.pntd.0007020

**Published:** 2019-02-21

**Authors:** Vivek B. Ravindran, Aravind Surapaneni, Nicholas D. Crosbie, Jonathan Schmidt, Esmaeil Shahsavari, Nagalakshmi Haleyur, Sarvesh K. Soni, Andrew S. Ball

**Affiliations:** 1 School of Science, RMIT University, Melbourne, Victoria, Australia; 2 South East Water, Frankston, Victoria, Australia; 3 Melbourne Water, Docklands, Victoria, Australia; PUCRS, BRAZIL

## Introduction

The human roundworm, *Ascaris lumbricoides*, is the major soil-transmitted helminth (STH) of public health concern, with an estimated prevalence of approximately 1.2 billion people, especially in Southeast Asia, Latin America, and sub-Saharan Africa [1]. Poor sanitation, poor hygiene, and inadequate water supply are considered the major epidemiological factors of ascariasis. The reuse of wastewater and sludge as an alternative to freshwater in agriculture is partly responsible for widespread STH infections [2]. A low infective dose combined with extreme resistance to environmental stress and excretion of up to 200,000 ova per day in the faeces of infected hosts enhances the infectivity of *Ascaris* [3].

In many countries with good sanitation systems (e.g., Australia), ascariasis is not endemic in humans and *Ascaris* ova are not commonly detected in raw sewage [4, 5]. However, increased migration from Asia and Latin America, in addition to the growing rates of travel to developing countries, is likely to increase the incidence of ascariasis [6]. Therefore, the removal of *Ascaris* ova from wastewater in developed countries remains crucial to allow the safe use of sludge or recycled water in agriculture [7]. For the unrestricted use of wastewater in agriculture, the World Health Organisation (WHO) has formulated stringent guidelines and has recommended ≤1 viable *Ascaris* ova/L for treated wastewater (recycled water) and ≤1 viable *Ascaris* ova/g dissolved solids for sludge to minimise public health risk associated with *Ascaris* species [8]. As a result, the methods utilised for enhanced ova recovery plays a significant role in accurately enumerating their presence in wastewater and sludge samples.

The United States Environmental Protection Agency (US EPA) has recommended the Tulane method for the detection and enumeration of STH ova in wastewater and sludge [9]. This method employs a series of steps, such as desorption, sedimentation, sieving, and flotation followed by enumeration using optical microscopy [10]. However, this method is time consuming because it takes approximately three days. Although various protocols have been developed to increase the recovery efficiency of *Ascaris* ova, until now there is lack of a universally acceptable method for the enumeration of STHs ova [11]. Therefore, the focus of this study was to develop a recovery method for *Ascaris* ova with minimal processing time and less damage to ova, without compromising the recovery efficiency and to compare the modified protocol with published methods.

## Methods

Samples of raw wastewater and sludge were collected from two different wastewater treatment plants (Lang Lang and Blind Bight) run by South East Water, Victoria, Australia. Because no indigenous *Ascaris* ova were observed in the aforementioned samples while analysed using the US EPA recommended Tulane method [10], the samples were spiked with *Ascaris suum* ova for the initial experiment investigating the impact of modifications in the Tulane method (Table 1) on the recovery efficiency of *A*. *suum*. The ova of *A*. *suum* were recovered from infected pig faecal samples and were used as a surrogate for *A*. *lumbricoides* due to their lower infectivity to humans, despite being identical morphologically and exhibiting 98.1% genetic similarity to *A*. *lumbricoides* [12, 13]. The ova were preserved in 5% potassium dichromate and stored at 4°C. A 1 mL aliquot of *A*. *suum* ova suspended in 1% phosphate buffered saline to a final concentration of 500 (±20) ova was used. Each recovery efficiency experiment was conducted in triplicate.

**Table 1:** Parameters of the original Tulane methods that have been modified during the development of an optimised protocol for the recovery of *Ascaris* ova.

Surfactant 1% 7× (Y/N)	Homogenisation (Y/N)	Settling time	Settling volume	Flotation step	Magnesium sulphate specific gravity	% Mean recovery efficiency
Y	Y	120	500	2 × 3	1.20	69
Y	Y	120	500	2 × 5	1.20	64
Y	Y	120	200	2 × 3	1.20	51
Y	Y	120	200	2 × 5	1.20	54
N	Y	120	500	2 × 3	1.20	19
N	Y	120	500	2 × 5	1.20	26
N	Y	120	200	2 × 3	1.20	21
N	Y	120	200	2 × 5	1.20	28
Y	Y	30	500	2 × 3	1.20	66
Y	Y	30	500	2 × 5	1.20	61
Y	Y	30	200	2 × 3	1.20	42
Y	Y	30	200	2 × 5	1.20	45
N	Y	30	500	2 × 3	1.20	28
N	Y	30	500	2 × 5	1.20	34
N	Y	30	200	2 × 3	1.20	25
N	Y	30	200	2 × 5	1.20	16

The yellow highlighted rows indicate that, based on the optimisation, those two parameters showed enhanced ova recovery.

**Abbreviations:** N, no; Y, yes.

The standard Tulane method [10] consisted of a settling volume of 900 mL, settling time of approximately 240 min with a single centrifugal flotation at 800 g for 10 min. Table 1 indicates the parameters that were modified from the original Tulane method in order to optimise parameters leading to an increased yield of ova recovery whilst minimising processing time. For homogenisation, a commercial mixer blender with 18,000 rpm at 1 min was used.

The percentage mean recovery of *Ascaris* ova was calculated as follows:

No. of ova recovered from spiked sample ÷ No. of ova spiked × 100 = % recovery

Based on the initial recovery efficiency experiment (Table 1), the highlighted parameters with the settling time of 30 min and 120 min showed enhanced recovery efficiency when compared with the original Tulane method. The results also highlight the importance of the addition of the surfactant 7× (1%) for enhanced ova recovery because it dissociates the *Ascaris* ova from the solid particles in wastewater and sludge. We selected a settling time of 30 min on the basis of reduced processing time.

The recovery efficiency of the modified method was then compared with the Tulane method [10] and our previously published double flotation method [14]. Raw wastewater (W) and sludge (S) samples were seeded with 1,000 (± 50) *Ascaris suum* ova for each sample. The parameters of the modified method differed significantly from the standard Tulane method and the double flotation method in all parameters except the homogenisation (18,000 rpm for 1 min) and the addition of the surfactant 7× (1%) for sedimentation (Table 2).

**Table 2:** Comparison of steps involved in each method to recover *Ascaris* ova.

Steps involved	Tulane method [10]	Double Flotation method [14]	Modified method
Settling volume	900 mL	900 mL	500 mL
Surfactant	1% 7×	1% 7×	1% 7×
Homogenisation	1 min	1 min	1 min
Settling time (mins)	240	240	30
Sieving	Yes	No	Yes
Flotation time (mins)	10	5	3
Flotation step	1	2	2
Specific gravity	1.20	1.25	1.20

## Results

**Fig 1:**
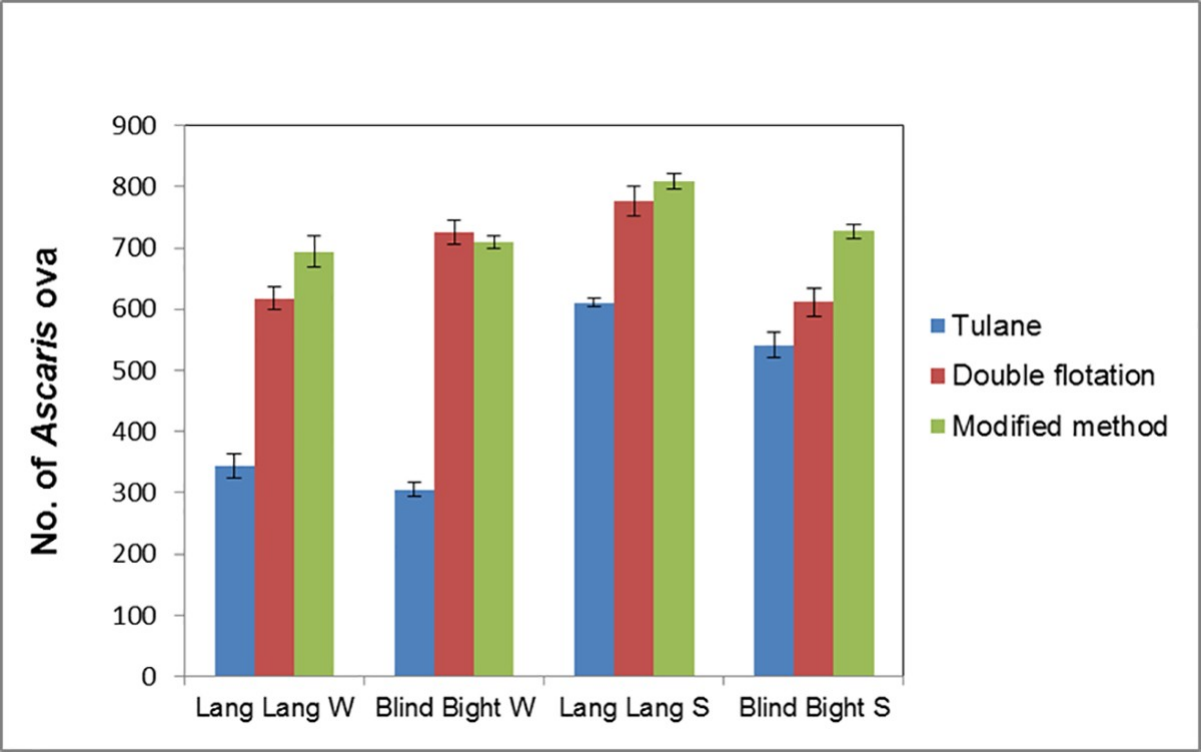
Comparison of maximum ova recovery efficiency of three different methods. The Tulane method (blue), double flotation method (red), and the modified method (green) were used for recovering *Ascaris* ova (seeded) from samples of wastewater (W) and sludge (S) from Lang Lang and Blind Bight Wastewater treatment plants. Modified method showed higher recovery of *Ascaris* ova compared to the other two methods.

The results shown in Fig 1 indicated enhanced *Ascaris* ova recovery from wastewater and sludge samples using modified method compared to the double flotation and the standard Tulane methods.

Overall an enhanced ova recovery was evident when samples were processed using the modified method. The modified method was statistically significant (*p* value <0.0001, paired T-test) denoting the accuracy in estimating the concentration of *Ascaris* ova from wastewater and sludge. This study presents an improved method for enumerating *Ascaris* ova in wastewater and sludge that is appropriate for resource-limited settings. This method is relatively fast; approximately 20 samples can be processed in a day. In comparison, the original US EPA method takes at least three days to complete approximately 10 samples (Box 1). The recovery efficiency was higher for the modified method that included a significant number of modifications from both the Tulane and double flotation methods.

**Box 1. Three advantages and three disadvantages of the new modified method**

Advantages

1. Reduced processing time with precision and accuracy,
2. Enhanced recovery efficiency due to minimal loss of ova by avoiding excessive contact with flotation solution and reagents,
3. Simple, reproducible, and cost-effective for routine use and in resource-limited settings to enumerate STH ova.

Disadvantages

1. Not tested with large sample volume;e,
2. Although the modified method requires minimal processing time than the other ova recovery methods, it is time consuming when compared with DNA-based point-of-care diagnostic devices;.
3. Need for instruments such as centrifuge and blender.

## Discussion

Our recovery experiments in the lab identified that using 1% 7× significantly (Table 1) increased the ova recovery efficiency. This is consistent with the standard US EPA method and a published method for enumerating *Ascaris* in hand rinsed samples [15]. Limbro 7× is an anionic, nonviscous surfactant that can effectively dissociate the bond between *Ascaris* ova and solid particles in wastewater and sludge. Bowman and colleagues (2003) compared the efficacy of the surfactants Limbro 7X, Triton X, and 0.1% Tween 80 in terms of the recovery of *Ascaris* ova. They reported increased recovery of ova recovery with 7×. In addition, 7× does not form a precipitate when in contact with the flotation solution. Additionally, homogenising the samples with a blender for 1 min enhanced the dissociation of ova from solid particles. However, increased homogenisation may reduce the viability of ova. The viability assessment was not performed in our recovery experiments.

Because the settling velocity of *Ascaris* ova in wastewater is 0.1582 mm/s [16], theoretically the settling time in a standard 1000 mL beaker for a settling volume of 500 mL is approximately 7 min and for 200 mL is approximately 3 min. Although there was little difference in the recovery, efficiency between the settling volumes utilised for the initial study, the difference in time between the two settling volumes can reduce the processing time. The effect of flotation solution on ova recovery efficiency was also evaluated. Because the specific gravity of *A*. *suum* is 1.13 [17], we observed that magnesium sulphate with the specific gravity of 1.20 was sufficient for the flotation of *A*. *suum* ova. However, magnesium sulphate with a specific gravity of 1.25 will be required for other STH ova, especially *TaeniaI*, which has a specific gravity of 1.23. Other STH ova require a flotation solution with an increased specific gravity of 1.30. In this experiment, the recovery efficiency was higher following 5 min of centrifugal flotation at 800 g. Increasing the contact time with the flotation solution to 10 min might damage the membrane integrity of ova. It has been reported that zinc sulphate is toxic to ova, and overnight soaking in magnesium sulphate may inactivate embryonated ova [10]. The presence of inhibitors in wastewater such as humic and fulvic acids can also have an impact on the recovery efficiency of ova.

In addition to the parameters tested during this study, there are several recovery parameters that have yet to be tested. Ova may be lost if they adhere to the walls of pipettes, tubes, and beakers. To reduce this possibility, some protocols recommend treating glassware with organosilane to reduce adhesiveness [10]. In contrast, it has also been reported that organosilane treatment reduced yields from both glass and plastic materials and recommended that noncoated pipettes and Falcon tubes be used in protocols to recover helminth ova [15]. Some protocols indicate preprocessing of samples by filtering via coarse or fine sieves prior to homogenisation and other steps in order to remove larger particles in wastewater [8]. Often, there is a risk of loss of ova associated with the discarded material. As a result, homogenisation and dissociation of ova from the matrix prior to filtration mitigates the risk.

## Conclusion

Despite the enhanced recovery efficiency with the modified method, these adaptations have to be validated according to a suitable Quality Assurance–Quality Control program, which would guarantee a more accurate enumeration and could result in the development of a universal method suitable for wastewater and sludge samples of diverse composition and origin.

## Supporting information

S1 Table Recovery of *Ascaris* ova using Tulane method, double flotation method, and modified method. (XLSX)
